# Characteristics of Excitable Dog Behavior Based on Owners’ Report from a Self-Selected Study

**DOI:** 10.3390/ani6030022

**Published:** 2016-03-16

**Authors:** Anastasia Shabelansky, Seana Dowling-Guyer

**Affiliations:** 1 Animal Rescue League of Boston, 10 Chandler Street, Boston, MA 02116, USA; 2 Center for Shelter Dogs, Department of Clinical Sciences, Cummings School of Veterinary Medicine, Tufts University, North Grafton, MA 01536, USA; seana.dowling_guyer@tufts.edu

**Keywords:** dog behavior, behavior problem, excitable behavior, owned dogs

## Abstract

**Simple Summary:**

This study provides information about owners’ experiences with their dogs’ excitable behavior. We found that certain daily scenarios tended to prompt excitable behavior. The majority of owners in this self-selected sample were very frustrated with their excitable dog. Many dogs in the sample had other behavior problems.

**Abstract:**

Past research has found that excitable dog behavior is prevalent among sheltered and owned dogs and many times is a reason for canine relinquishment. In spite of its prevalence in the canine population, excitable behavior is relatively unstudied in the scientific literature. The intent of this research was to understand the experience of owners of excitable dogs through the analysis of self-administered online questionnaires completed by owners as part of another study. We found that certain daily scenarios tended to prompt excitable behavior, with excitability most common when the owner or other people came to the dog’s home. All owners experienced some level of frustration with their dog’s excitable behavior, with the majority being very frustrated. Many dogs in the sample had other behavior problems, with disobedient, destructive, chasing and barking behaviors being the most commonly reported. Other characteristics of excitable dogs also are discussed. Although the ability to generalize from these results is likely limited, due to targeted recruitment and selection of owners of more excitable dogs, this research provides valuable insights into the owner’s experience of excitable behavior. We hope this study prompts more research into canine excitable behavior which would expand our understanding of this behavior and help behaviorists, veterinarians, and shelters develop tools for managing it, as well as provide better education to owners of excitable dogs.

## 1. Introduction

Excitable dog behavior such as jumping on people is a very common behavior, especially for young dogs [1,2,3]. A recent study reported jumping up as the most common behavioral complaint (56.8%) described by dog owners who attended the Small Animal Hospital, University of Tehran [4]. This behavior can be bothersome and sometimes even dangerous, particularly when the dog is large. Excitable behavior is often difficult to stop and is exhibited in numerous daily routine scenarios such as greeting, playing, requesting food, or meeting someone [3].

Although excitable behavior seems prevalent in both owned and sheltered canine populations [2,5], there is no accepted definition in the scientific literature for it. Excitable behavior may be seen as ranging from jumping on people to nipping and even biting. It is also unclear what behaviors should be categorized under excitable behavior, what behaviors contribute or coexist with it, and what situations provoke it. In the principal component analysis performed by Bennett and Rohlf [6], jumping on people loaded together with pulling on the leash and engaging in inappropriate sexual behavior. Landsberg *et al.* [2] distinguish between jumping up on people *versus* hyperactivity and unruliness, which include: extensive jumping up, nipping and biting, destructiveness, digging, and some forms of barking. Landsberg *et al.* [2] also point out that overactive or unruly dogs respond poorly to commands and it is hard to control their behavior. Khoshnegah *et al.* [1] and Wells and Hepper [3] use the term “excessive activity” to describe dogs with similar behavioral characteristics. According to Khoshnegah *et al.* [1], excessively active dogs exhibit a range of behaviors: they move constantly and quickly, run and jump, demonstrate an excessive degree of restlessness, and have difficulty adjusting to new surroundings. The authors also classify destructiveness and excessive barking as separate behavior problems that may or may not occur with excessively active behavior. The Center for Shelter Dogs [7] describes excitable dogs as exhibiting one or more of the following behaviors: energetically jumping up, putting their mouths on people with some degree of pressure (usually people’s arms and legs), and grabbing at clothing and/or the leash. Although the definitions presented above have some degree of consistency, they are not entirely in agreement on the characteristics of excitable behavior.

Many times, a dog’s problem behavior has detrimental effects on the relationship between it and its owners, and is a common reason for canine relinquishment [8,9,10]. Even though excitable behavior is a relatively “normal” behavior in juvenile dogs, it would appear that many owners regard it as undesirable [3], especially in adult dogs. Hyperactive behavior was reported by 53% of people relinquishing their dog to a shelter [11]. Disobedience was cited as a primary reason for relinquishment by 13% of dog owners [12]. Yet, few studies have investigated in any detail the owner’s experience of their dog’s excitable behavior or the role that it plays in the owner’s decision to relinquish.

Previous studies have found an association between specific pet and owner characteristics and excitable behavior [13,14]. A statistically significant association was found between first-time ownership and the prevalence of various manifestations of overexcitability [13]. In addition, there is evidence of an association between over-excitement and displacement activities in the dog and anxiety in the owner [14]. Although these studies examined the association between owner characteristics and certain excitable behaviors in dogs, none investigated the owners’ experience with their excitable dogs. Information about the owners’ experience with excitable behavior, its characteristics, as well as what may routinely prompt it can help researchers gain a better understanding of excitable behavior. Moreover, since the scientific community lacks a standard definition of excitable behavior, owners of excitable dogs present a prime opportunity to launch research into dogs’ excitable behavior. Finally, shelters, veterinarians, and behaviorists can obtain useful information in order to successfully place these animals into suitable homes and design appropriate behavior modifications or training plans for excitable dogs.

In the current study, a self-selected sample of owners of excitable dogs, generated for another study, was utilized. The main objective was to analyze the data reported by owners, specifically concentrating on the following topics: 1. the excitable dogs’ behavior at home, around their owner; 2. the behavior of these dogs around new people and in a new environment; 3. the behavior of excitable dogs around other canines; 4. the owners’ perception and tolerance of excitable behavior in relation to its severity; 5. other behavior problems that may coexist with excitable behavior. Owners rated the frequency of specific excitable behaviors in several, common scenarios. In these scenarios, excitable behavior was defined as uncontrollably jumping up, mouthing with no discomfort (soft mouthing), mouthing with discomfort (hard mouthing), and grabbing clothes (or leash during walks). In other questions, owners were asked to describe their dogs’ excitable behavior in an open-text response question, in order to gain insight into the way owners define and experience this behavior.

## 2. Methods

### 2.1. Owner Survey

This study was part of a broader project carried out at an animal shelter in Massachusetts, US in order to identify the effectiveness of a behavior modification training plan for excitable dogs. The survey results were originally used as selection criteria for the project and were not reported elsewhere. The survey obtained a large amount of information, some of which was irrelevant to the current study. The focus of this study is only on owners’ experience with their excitable dogs.

Between September and November 2012, a recruitment ad for the survey was posted on a local Boston newspaper website, blogs and several social media websites. In addition, employees of two urban shelters in Massachusetts, US, were asked to forward the online survey link to people who may have highly excitable dogs. Owners were allowed to participate in the survey based on a subjective definition of their dogs’ excitable behavior. The recruitment criteria were: highly excitable dogs who are difficult to manage due to their excitement and activity level, 6 months of age or older, weighing more than 11 kg, healthy, up to date on vaccinations, and able to participate in walks and exercise. If the owner’s answers did not match the requirement criteria (owner description of their dog as highly excitable or highly energetic, 11 kg or more, up to date on vaccinations, and ability to participate in walks and exercise), the owner was automatically terminated from the survey. Weight (11 kg) was a recruitment criterion for the broader project, selected since, anecdotally, excitable behavior is more problematic to the owner when the dog is larger.

The questionnaire contained five sections. The first section included questions about the dog’s age, weight, vaccination records and familiarity with the site of the broader project, a boarding facility. The second section of the questionnaire inquired about the dog’s reactions to other dogs and the dog’s play style with other dogs, information which was important for use at the boarding facility. Using a closed format question, owners indicated specific behavioral reactions to other dogs (e.g., bark, growl, lunge). In the open-response question, owners were asked to describe how their dog likes to play with other dogs. For example, if the dog plays rough, likes to chase or be chased, likes to be around other dogs but not play, *etc.* The third part of the questionnaire asked about the dog’s behavior around the owner, and focused on the excitable behavior at home. In the beginning of the third section, all owners were asked in a closed format question if they would describe their dog as highly excitable or highly energetic. If owners answered “no” on this question they were not allowed to complete the survey and, therefore, were not included in this study. All owners who answered “yes” on this question then were asked if they found the excitable behavior difficult to manage, followed by questions where they rated how excitable their dog is and how frustrated they are with their dog’s excitable behavior. A 5 point Likert-type scale was utilized: a rating of 1 indicated “not excitable/frustrated at all” and a rating of 5 indicated “extremely excitable/frustrated”. At the end of the section, all owners were asked to describe their dogs’ behavior in a new environment and around new people in an open-response text field. In the fourth part of the survey, owners were asked about their dog’s excitable behavior in specific situations common to daily living: when they arrived home after an absence, prepared to feed the dog, picked up the leash to take the dog outside, rested or watched TV, and played with the dog. In all scenarios, owners indicated the frequency of specific, individual excitable behaviors (jumping up on a person, putting mouth on owner’s body causing no discomfort, putting mouth on owner’s body causing discomfort, grabbing clothes, and grabbing a leash) using the following scale: never, rarely, sometimes, frequently, and always. In addition, a “don’t know/” option was included for all questions. All owners were asked to provide information about any behavior problems that they were concerned about in an open-response text field. Finally, in the fifth section of the survey, owners provided additional demographic information about their dog such as sex, neuter status, and breed. Unfortunately, owner demographic data were not collected since that was not relevant to the objective of the broader study.

### 2.2. Statistical Analysis

Descriptive statistics were generated and data were analyzed using IBM SPSS Statistics, version 20 (Armonk, NY, USA). Frequencies of each behavior were reported for every scenario. For tests of association, Chi-Square was calculated to identify the relationship between categorical data. The Mann-Whitney U test was used when the dependent variable was ordinal, there were only two groups of the independent variable, and the samples were independent. The Kruskal-Wallis test was performed when the dependent variable was measured on an ordinal scale, there were more than two groups of the independent variable, and the samples were independent. The Friedman test was used when the dependent variable was ordinal and the samples were dependent. Z scores were used to detect differences between proportions. Open-ended questions were analyzed using a semantic approach; answers were grouped into themes with percentages based on the total sample reported. For all analyses, an alpha level of 0.05 was used to determine statistical significance, although the significance level was adjusted for multiple comparisons in post-hoc tests using the Bonferroni approach.

## 3. Results and Discussion

### 3.1. Dogs’ Demographic Information

A total of 182 owners completed the survey, seven of whom answered that they did not consider their dog to be highly excitable and energetic and whom, consequently, were excluded from the study sample. This resulted in a final sample of 175 owners of highly excitable dogs.

Based on owners’ reports, of the 175 dogs included in the study, four (2.3%) were intact females, 74 (42.3%) were spayed females, eight (4.6%) were intact males and 89 (50.8%) were neutered males. More owners reported their dogs to be mixed breed (91, 52.0%) than purebred (80, 45.7%), with four (2.3%) owners unsure of their dogs’ breed. The most common breeds for purebred dogs were: Labrador Retriever (14/80, 17.5%), Pit Bull (8/80, 10.0%), and German Shepherd (5/80, 6.3%). Owners of mixed breed dogs were asked to specify what breed their dog looked like. For mixed breed dogs, the most common predominant breeds were: Labrador Retriever (24/91, 26.4%), Pit Bull (12/91, 13.2%), Boxer (8/91, 8.8%) and German Shepherd (7/91, 7.7%). Mean weight and age (±SD) were 26.6 ± 9.9 kg and 3.1 ± 2 years, respectively. Sixty-six dogs (48%) lived with another dog in the house, and 15 (23%) of them lived with two or more dogs.

### 3.2. Frequency and Intensity of Excitable Behavior around the Owner, Reported in Closed Survey Scenarios

Table 1 shows total frequency and intensity of excitable behavior toward the owner. As can be seen, total frequency and intensity of excitable behavior varied among scenarios. Overall, “jump up” and “put mouth on body causing no discomfort” were more commonly reported than “put mouth on body causing discomfort” or “grab clothes”. Two scenarios triggered the highest rates of excitable behavior: when the owner came home and when the owner played with the dog. Approximately 60% of dogs frequently or always jumped up in these scenarios, 40% frequently or always put their mouths on a person’s body but caused no discomfort, and 15% frequently or always grabbed a person’s clothes with their teeth. Play with owners also triggered the highest rates of mouthing with discomfort: 11% of dogs frequently or always put their mouths on a person’s body causing discomfort in this scenario. Only about 6% of dogs in the sample never jumped up in either of these two scenarios. Mealtimes did not trigger a lot of excitable behaviors: nearly 70% of owners indicated that their dog never or very rarely jumped up when they were preparing food for their dog.

Across scenarios, the Friedman test revealed statistically significant differences in the frequencies of some behaviors: jumping up (*X^2^*(4) = 277.23, *p* < 0.001), mouthing with no discomfort (*X^2^*(4) = 321.91, *p* < 0.001), mouthing with discomfort (*X^2^*(4) = 161.32, *p* < 0.001), and grabbing clothes (*X^2^*(4) = 192.57, *p* < 0.001). Results from post-hoc tests of the pairwise differences amongst the groups, using the Bonferroni approach to control for Type I error, are reported in Table 1.

Within each scenario, the Friedman test revealed statistically significant differences in the frequencies of some behaviors: owner coming home (*X^2^*(3) = 260.63, *p* < 0.001), owner resting or watching TV (*X^2^*(3) = 192.62, *p* < 0.001), owner playing with dog (*X^2^*(3) = 277.16, *p* < 0.001), preparing meal (*X^2^*(3) = 140.46, *p* < 0.001), and picking up leash (*X^2^*(3) = 262.04, *p* < 0.001). Results from post-hoc tests of the pairwise differences amongst the groups, using the Bonferroni approach to control for Type I error, are reported in Table 1.

There were few relationships between a dog’s demographics and owner excitability and frustration ratings. The Mann-Whitney U test revealed a non-significant difference between the dog’s sex and owner excitability rating (*p* > 0.05) and owner frustration rating (*p* > 0.05). However, owners rated spayed or neutered dogs as significantly less excitable (Mdn = 4) than unspayed or unneutered dogs (Mdn = 5), as determined by a Mann-Whitney U test (*U* = 1292.00, *p* = 0.04). According to a Mann-Whitney U test, the difference in owner frustration ratings by spay/neuter status was not significant (*p* > 0.05). Using Spearman’s rho for ordinal data, a weak but significant correlation was found between dog’s age and owner excitability rating (*r* = −0.18, *p* = 0.04) but not between age and owner frustration rating (*p* > 0.05). Looking at the dog’s weight, there were weak but significant relationships between weight and owner excitability rating (*r* = −0.18, *p* = 0.02) and weight and owner frustration rating (*r* = 0.16, *p* = 0.04).

### 3.3. Behavior of Excitable Dogs around New People and in a New Environment

Although owners were asked about their dog in a new environment in an open-ended question, many of them described situations when new people came to their house. The qualitative analysis of this question revealed that the majority of owners (114, 65.1%) described their dog as being very comfortable around new people and in new environments. “Excited” (31, 17.7%) and/or “curious” (15, 8.6%) were the most commonly used adjectives to describe the dog’s behavior. Behaviors that were frequently mentioned were: jumps, plays, wags, licks, sniffs, barks. Fifteen (8.6%) owners described their dogs as initially shy when introduced to a new environment or new people but comfortable after some time. For nearly 11% (19) of owners, the dog’s behavior had a situational context such as, “He can be totally fine with new people but he can also be aggressive…he likes being approached on his own terms.” Other variables that had a potential impact on these dogs’ behavior included the way a stranger approached the dog, the stranger’s gender, environmental triggers such as sound, or the presence of another dog. Nearly 8% (13) of owners described their dog as being intimidated or nervous in new environments and/or around new people, reporting, “My dog is nervous around adults and barks and runs away from people when they enter our house. She is actually better around other people outside of our home but is still scared of them”. Barking was reported in these situations as well. Owners of dogs they described as intimidated or nervous in new environments and/or around new people also reported their dogs experiencing separation anxiety when left alone in a new environment.

### 3.4. Behavior of Excitable Dogs around Other Dogs

Forty-three percent of owners (75) reported that their dog typically shows one or a combination of the following aggressive reactions when he/she sees other dogs on- or off-leash or both: growls/shows teeth, snaps/lunges, barks aggressively. Twenty-three percent (41) of them reported that their dog had shown aggression toward both on-leash and off-leash dogs. “Barks aggressively” was the predominant aggressive behavior in both categories (Table 2).

Z scores revealed the proportion of dogs reported by owners to bark aggressively at other dogs while on leash was significantly higher than those reported to growl or show teeth (*z* = −5.14, *p* < 0.01) or snap or lunge (*z* = −2.09, *p* = 0.04). The proportion of dogs reported to snap or lunge at other dogs while on leash was significantly higher than that reported to growl or show teeth (*z* = −3.23, *p* < 0.01).

A similar pattern emerged when looking at off leash behavior. Z scores revealed the proportion of dogs reported by owners to bark aggressively at other dogs while off leash was significantly higher than those reported to growl or show teeth (*z* = −4.03, *p* < 0.01) or snap or lunge (*z* = −2.31, *p* = 0.02). The proportion of dogs reported to snap or lunge at other dogs while off leash was not significantly different than that reported to growl or show teeth (*z* = −1.86, *p* < 0.06).

There were no significant differences when looking at the proportion of dogs exhibiting each behavior at other dogs on *versus* off leash (*p* > 0.05).

### 3.5. Play Style with Other Dogs

Thirteen percent (23) of owners skipped the open-ended “play style” question. Of those who responded, some owners used precise terms to describe their dog’s play style such as, “wrestles”, “chases”, or “runs around” while others simply stated that the dog is “rough” or “plays well”. Forty-six percent (81/175) of owners reported that their dog’s favorite play style was to chase other dogs, be chased, or both. Other commonly mentioned favorite play styles were running (22, 12.6%) and wrestling (18, 10.3%). Nearly 25% (43) of owners reported their dog being rough while playing with other dogs, as in, “[My dog] is affectionately known as a “body slammer”. He is high-energy, rough, and fast”. Jumping up, mouthing/nipping and different vocalizations during play were also reported. Only a few dogs were reported to be scared or aggressive, and, consequently, could not play with other dogs. About 10% (17) of owners reported that their dogs loved to play, but not with every dog. Familiarity with the other dog and its behavior as well as its size were factors. According to one owner, “[It] depends on the dog, with some he will rough house… with others he will not play at all. He seems to be nervous around larger dogs”.

### 3.6. Owners’ Perception of Excitable Behavior

All owners rated their dogs as a 3 or higher on the question about the severity of their dog’s excitability (e.g., “Please rate how excitable your dog is”, with 1 being “Not excitable at all” and 5 being “Extremely excitable”): 14 (8.0%) rated their dog a 3, 92 (52.6%) rated their dog a 4, and 69 (39.4%) rated their dog a 5. In addition, the majority were frustrated with their dog’s excitable behavior (e.g., “Please rate how frustrated you are with your dog’s excitable behavior”, with 1 being “Not frustrated at all” and 5 being “Extremely frustrated”): 19 (10.9%) rated their frustration a 2, 56 (32.0%) rated a 3, 61 (34.9%) rated a 4, and 39 (22.3%) rated a 5. There was a statistically significant difference between the excitability ratings and owners’ ratings of frustration (*H*(2) = 20.14, *p* < 0.001). Owners of less excitable dogs felt less frustrated (mean rank frustration rating for excitable rating of 3 = 41.3, mean rank frustration rating for excitable rating of 4 = 84.0, mean rank frustration rating for excitable rating of 5 = 102.8). Post-hoc tests of the pairwise differences amongst the groups, using the Bonferroni approach to control for Type I error, revealed all three groups (excitability ratings of 3, 4, and 5) were statistically different from each other.

Almost all owners (156, 89.1%) found the excitable behavior difficult to manage. Owners who said that the behavior was not difficult to manage were less frustrated with their dogs (*X*^2^ = 45.04, *p* < 0.001), and also reported their dogs were less excitable (*X*^2^ = 13.91, *p* < 0.001).

### 3.7. Other Behavior Problems Reported to Coexist with Excitable Behavior

Eleven people (6%) skipped the open-ended question about other behavior problems entirely. For the rest, although they were asked about other behavior problems, many of them felt the need to reiterate and provide a further explanation of their dog’s excitable behavior. About 22.9% (40/175) of owners were concerned about their dog’s excitable behavior when meeting people. Concerns varied from the dog’s jumping or putting its mouth around an arm to nipping and grabbing clothes. The majority (32/40, 80.0%) of these respondents described their dog as jumping excessively on visitors or new people. According to one owner, “He lunges, licks, [and] gives them no personal space.” Another owner described even more severe behavior, “He barks and can jump up in the air to reach people, children’s faces... He bites their clothes or hands or arms.” Disobedient and destructive behaviors were also commonly reported by owners, ranging from pulling on the leash (35, 20.0%) to climbing furniture (11, 6.3%) and destroying household items (14, 8.0%). Some owners specified that their dog does not listen or respond to commands (22, 12.6%), with examples such as, “My dog jumps on [the] kitchen counter with all four feet to steal cat food… almost never listens, destroys toys…jumps over [the] gate, steals from [the] trash”. Another significant theme that emerged from owner responses was chase behavior (17, 9.7%). The dogs were chasing children, small animals (such as cats, small dogs, and squirrels), and moving objects (such as skateboards, bikes, cars, floor cleaners, joggers). One owner reported, “If off leash he has a habit of suddenly deciding to chase cars (he used to chase anything that moved faster than a walk when we first got him—joggers, bikes, *etc.*).” Barking behavior was another theme that emerged, with the dogs barking at people (23, 13.1%), other dogs (12, 6.9%), or the front door or window. The nature of the barking behavior varied; according to owners, some dogs barked due to overexcitement, and in some cases barking turned into more severe behavior. “When she gets overstimulated, she barks at me”, one owner reported, “and grabs and shakes my shoelace or pants leg or leg (sometimes breaking the skin). Overstimulation can come from a sneeze from me on a hike, or whenever we turn back on a walk/hike, if I stumble or fall, or reasons I can’t explain…She can be so frustrating”. Some barking behavior had a protective nature. “When he sees someone outside our house, he charges at the window and barks extremely loud,” one owner noted. Some barking behavior was exhibited primarily outside when the dog was walked on a leash. “When walking him, he barks at strangers uncontrollably and will not stop until they pass by…even if he knows the person, he acts like he is going to attack them. He will be aggressive if someone jumps nearby or [if] kids are playing roughly he will become aggressive.” Although it was very hard to interpret the owners’ responses if the dog was described as aggressive or as overstimulated and excessively excitable without any specific behaviors included in the description, some owners (11, 6.3%) described their dogs as being sometimes aggressive to people or mentioned the dog’s biting history: “He can be aggressive if he feels uncomfortable or overwhelmed”, “His major issue is his reaction when he sees another animal that he cannot get …He turns his frustrations on anyone and anything that is near him, biting and barking uncontrollably”. Aggression directed toward other dogs (13, 7.4%) was slightly more common than aggression toward people. Anxiety, digging, and escaping behaviors were rarely mentioned.

### 3.8. Discussion

In this study, the survey responses of owners of excitable dogs were analyzed in order to explore and understand their experiences with their dogs. The findings demonstrate that excitable behavior is indeed complex, with a range of severity and frequencies, prompted by many daily scenarios. However, excitability was most commonly reported, with greater frequency and more severity, when the owner or other people came to the dog’s home or played with the dog. Playtime in particular triggered more severe behavior which included hard mouthing of body parts. This may be due to the stimulation that usually comes with “play” such as loud voices, fast movements, and toys. Surprisingly, food preparation by the owner did not prompt excitable behaviors, with less than a third of owners reporting jumping some of the time or more frequently. This may be due to effective owner modification or management of the behavior, where owners could have trained their dogs to sit and wait for the food. Owners could have also prepared the food in a different room without the dog seeing it.

All owners experienced some level of frustration with their dog’s excitable behavior, with the majority being very or extremely frustrated. In addition, the more excitable the dog was, the more frustrated the owner was, although it is possible more frustrated owners perceived their dog’s behavior as more excitable. Not surprisingly, attention-seeking activity or general excitability of the dog could have become an irritant to the owner [9]. Although previous research found that owners consider excitable behavior undesirable [3], the self-selection bias impacted this study’s results. Perhaps the decision to participate in the study reflected some inherent bias in the characteristics of the participants. Presumably, a high frustration level would have contributed to the owner’s decision to enroll their dog in this study and, therefore, these results may not apply to a general population of excitable dogs’ owners. Unfortunately, due to the nature of this study, owner demographics were not collected. For future research, it would be interesting to explore if there is a relationship between the owner’s demographic characteristics such as age, gender, number of family members, other pets, housing environment, or daily activity, and excitable behavior of the dog. Moreover, it is possible that something in the owner behavior could provoke the excitable behavior of the dog. An in-depth survey or qualitative interviews of owners which ask about their care and interactions with the dog, such as play style, activity, reactions when the dog shows excitable behavior, *etc.* could provide more information about the etiology of excitable behavior.

In general, disobedient, destructive, chasing and barking behavior problems were the most commonly reported behaviors by owners of excitable dogs when asked about other behavior problems. Since these behaviors were frequently reported by our sample of owners, it would be important to include them in future research about excitability. Pulling on the leash also was frequently reported in our study. In previous research, this behavior was grouped together with jumping up in a data reduction technique [6], again suggesting that any future research about excitability should expand beyond the usual definition of excessive jumping and mouthing. If these findings replicate, they could be used to educate potential adopters of excitable dogs. Aggressive behaviors toward people and dogs also were reported. However, reporting of aggressive behavior was based solely on the owners’ individual understanding of it, and, therefore, it is possible that some owners could misinterpret behaviors such as hard mouthing as aggression. It is important to collect information about aggressive behavior as objectively and neutrally as possible, avoiding lingo and providing clear descriptions of the behavior, similar to what was used in the dog-to-dog aggression questions in this study. Unfortunately, in this study, we did not collect information about aggressive behavior toward people, only other dogs, as dictated by the requirements of the broader study.

The majority of dogs in the sample were reported to feel comfortable in a new environment and around new people. However, the fact that 41% of dogs in this sample had previously stayed in the boarding kennel, the site of the broader study, could have made them more comfortable than the average dog in new environments. Although 43% of owners reported their dog having a certain level of aggression toward other dogs, only a few of them described their dog as aggressive or fearful when their dog was playing with conspecifics. Previous research has reported that a dog’s reaction to other canines may vary based on the other canine’s behavior, sex and appearance [15]. We also found that, in an open-response question, some owners reported that their dog’s “play behavior” had a situational character: some dogs liked same-sized dogs, some preferred dogs of a certain personality, and the environment made the difference for others.

There were weak but significant relationships between some demographics of the dog and excitability. Although the negative relationship between age of the dog and excitability was expected, and the negative relationship between weight and excitability might make sense anecdotally, the weak relationship suggests these findings are unstable, at least in this study. Future research should consider these characteristics closely to determine if they are related to the development or expression of excitability, to what degree, and how they might moderate an owner’s perception of the behavior.

## 4. Conclusions

In conclusion, the large number of people who responded to this survey in a very short time with minimal advertising suggests that there is a need to bring more attention to excitable dog behavior. This study provides—for the first time—information about owners’ experiences with dogs’ excitable behavior, based on owners’ perception and understanding of it. Characteristics of excitable behavior, as well as what may routinely prompt it, were also presented. Future research should explore what owner-related factors contribute to excitable behavior. Since this study’s results were subjected to a self-selection bias, a nationally representative sample of owners could extend the research about excitable dogs. Moreover, such a sample would provide comparisons between excitable and non-excitable dogs, as defined by their owners. The last will help to clarify the definition of excitable behavior. In addition, in-depth qualitative interviews would provide a deeper understanding of not only excitable dogs’ behavior but also insight into owners’ experiences with and feelings about it. In-depth research into excitable behavior etiology as well as ethology would extend our understanding of this behavior. The research into excitable behavior could help behaviorists, veterinarians, and shelters to develop better tools for managing it, as well as provide better education to owners of excitable dogs, resulting in satisfying bonds between owners and dogs and successful long-term retention in the home.

## Figures and Tables

**Table 1:** Frequency and intensity of excitable behavior around the owner.

	Never		Rarely		Sometimes		Frequently		Always		Don't know		Within Scenario Differences	Between Scenario Differences
	N	%	N	%	N	%	N	%	N	%	N	%		
*How frequently does your dog show any of the following behaviors when you come home?*														
A1. Uncontrollably Jumps Up	11	6.3	22	12.6	37	21.1	49	28.0	56	32.0	0	0	A2, A3, A4	B1, D1, E1
A2. Puts mouth on your body causing **no** discomfort	34	19.4	26	14.9	49	28.0	38	21.7	27	15.4	1	0.6	A1, A3, A4	B2, D2, E2
A3. Puts mouth on your body causing discomfort	108	61.7	46	26.3	11	6.3	8	4.6	2	1.1	0	0	A1, A2, A4	C3, D3
A4. Grabs your clothes	83	47.4	26	14.9	40	22.9	19	10.9	7	4.0	0	0	A1, A2, A3	B4, D4, E4
*How frequently does your dog show any of the following behaviors when you rest or watch TV?*														
B1. Uncontrollably Jumps Up	38	21.7	26	14.9	66	37.7	32	18.3	12	6.9	1	0.6	B3, B4	A1, C1, D1, E1
B2. Puts mouth on your body causing **no** discomfort	55	31.4	33	18.9	48	27.4	26	14.9	11	6.3	2	1.1	B3, B4	A2, C2, D2
B3. Puts mouth on your body causing discomfort	120	68.6	34	19.4	12	6.9	6	3.4	1	0.6	2	1.1	B1, B2	C3, D3
B4. Grabs your clothes	100	57.1	35	20.0	26	14.9	11	6.3	2	1.1	1	0.6	B1, B2	A4, C4, D4
*How frequently does your dog show any of the following behaviors when you play with your dog?*														
C1. Uncontrollably Jumps Up	10	5.7	8	4.6	53	30.3	52	29.7	52	29.7	0	0	C2, C3, C4	B1, D1, E1
C2. Puts mouth on your body causing **no** discomfort	15	8.6	18	10.3	71	40.6	47	26.9	23	13.1	1	0.6	C2, C3, C4	B2, D2, E2
C3. Puts mouth on your body causing discomfort	83	47.7	49	28.0	22	12.6	17	9.7	3	1.7	1	0.6	C1, C2	A3, B3, D3, E3
C4. Grabs your clothes	70	40.0	34	19.4	41	23.4	23	13.1	6	3.4	1	0.6	C1, C2	B4, D4, E4,
*How frequently does your dog show any of the following behaviors at mealtimes when you are preparing to feed him/her?*														
D1. Uncontrollably Jumps Up	88	50.3	35	20.0	25	14.3	16	9.1	11	6.3	0	0	D2, D3, D4	A1, C1, D1, E1
D2. Puts mouth on your body causing **no** discomfort	131	74.9	28	16.0	11	6.3	2	1.1	2	1.1	1	0.6	D1	A2, B2, C2, E2
D3. Puts mouth on your body causing discomfort	152	86.9	18	10.3	4	2.3	1	0.6	0	0.0	0	0	D1	A3, B3, C3
D4. Grabs your clothes	144	82.3	18	10.3	9	5.1	1	0.6	0	0.0	0	0	D1	A4, B4, C4
*How frequently does your dog show any of the following behaviors when you pick up the leash to take your dog outside?*														
E1. Uncontrollably Jumps Up	31	17.7	18	10.3	45	25.7	39	22.3	42	24.0	0	0	E2, E3, E4	A1, C1, D1, E1
E2. Puts mouth on your body causing **no** discomfort	80	45.7	32	18.3	40	22.9	13	7.4	9	5.1	1	0.6	E2, E3, E4	A2, C2, D2
E3. Puts mouth on your body causing discomfort	127	72.6	33	18.9	10	5.7	5	2.9	0	0.0	0	0	E1, E2	C3
E4. Grabs your clothes	120	68.6	29	16.6	14	8.0	7	4.0	4	2.3	1	0.6	E1, E2	A4, C4

Note: Within and between scenario differences are statistically significant at the 0.05 level, or as adjusted by the Bonferroni approach.

**Table 2:** Reaction to other dogs on and off leash.

	1. Growls/Shows Teeth		2. Snaps/Lunges		3. Barks Aggressively		None of listed		Total	Within Scenario Differences
	N	%	N	%	N	%	N	%	N	
A. Does your dog typically show any of the following reactions when he/she sees other dogs ON leash?	10	5.7	29	16.6	45	25.7	107	61.1	175	A1 from A2, A1 from A3, A2 from A3
B. Does your dog typically show any of the following reactions when he/she sees other dogs OFF leash	8	4.6	17	9.7	32	18.3	127	72.6	175	B1 from B3, B2 from B3

Note: Multiple response table except “none of listed” which is mutually exclusive. Within scenario differences are statistically significant at the 0.05 level, or as adjusted by the Bonferroni approach.
